# Membrane-Based Pulsed Sampling Method for Extended Dynamic Range of Ion Mobility Spectrometry

**DOI:** 10.3390/s24103106

**Published:** 2024-05-14

**Authors:** Xinzhi Chen, Wencheng Lu, Di Lan, Bo Zhang, Hao Gu, Mutong Shen, Lingfeng Li, Peng Li

**Affiliations:** 1 School of Electronic and Information Engineering, Soochow University, Suzhou 215006, China; 2 Suzhou Weimu Intelligent System Co., Ltd., Suzhou 215006, Chinalingfengli@suda.edu.cn (L.L.)

**Keywords:** ion mobility spectrometry (IMS), membrane, dynamic range, sample introduction

## Abstract

Ion mobility spectrometry (IMS) has been widely studied and applied as an effective analytical technology for the on-site detection of volatile organic compounds (VOCs). Despite its superior selectivity compared with most gas sensors, its limited dynamic range is regarded as a major drawback, limiting its further application in quantitative measurements. In this work, we proposed a novel sample introduction method based on pulsed membrane adsorption, which effectively enhanced IMS’s ability to measure analytes at higher concentrations. Taking N-methyl-2-pyrrolidone (NMP) as an example, this new sampling method expanded the dynamic range from 1 ppm to 200 ppm. The working principle and measurement strategy of this sampling method were also discussed, providing new insights for the design and application of IMS-based instruments.

## 1. Introduction

The need to measure and monitor hazardous chemicals is becoming a critical issue as industrial manufacturing activities increases. In particular, volatile organic compounds (VOCs), including hydrocarbons, alcohols, aldehydes, and ketones, have drawn considerable attention due to their wide presence in the working environment of many industrial sectors, such as paints, detergents, fuels, electrolytes, etc. [1,2]. The emission of VOCs does not only jeopardize the health of workers [3,4] but also causes long-term impacts on the environment [5]. Therefore, on-site and real-time monitoring of concentration levels of VOCs is of great importance.

A number of existing technologies can be used for the monitoring of VOCs, including semiconductor gas sensors, the photon ionization detector (PID), and the flame ionization detector (FID). The working principle of semiconductor sensors relies on the adsorption of gas molecules on the surface of the sensitive layer, and this process triggers an electrical response that results in a significant change in resistance [6]. Various engineered materials have been applied to detect VOCs in the environment, including the metal oxide semiconductor (MOS) [7], conducting polymers (CPs) [8], and carbon-based nanomaterials (CNMs) [9]. The PID detects chemicals in the gas phase based on the ionization of VOC molecules through ultraviolet radiation, which requires that the ionization potential of the analyte is lower than the photon energy of the lamp used [10]. The FID is a device that ionizes target analytes through hydrogen flame and quantifies the concentration of ions according to the intensity of the ion current [11].

Although the three methods for detecting VOCs have good performance in terms of sensitivity, limit of detection, and dynamic detection range [12,13], they all suffer from poor specificity. The response is a weighted sum of all of the responsive chemicals in the introduced sample, and the sensor is unable to specify individual compounds from com-plex mixtures. Also, baseline drift due to prolonged exposure to VOC vapors introduces extra uncertainty in the measurement [14].

Other standard analytical chemistry technologies, such as Gas Chromatography (GC) and mass spectrometry (MS), are powerful in their capability for qualitative and quantitative measurement, but the instruments are normally bulky, expensive, and time-consuming and complicated to operate, which limits their applications to laboratory-based analysis rather than on-site monitoring [15,16].

Ion mobility spectroscopy (IMS) separates ionized molecules in the gas phase by their velocity in a defined electric field under atmospheric pressure, and it characterizes each ion species by its mobility and the amplitude of the ion current. Due to its compact size and fast analysis time, together with its high sensitivity and specificity, IMS represents a suitable tradeoff between simple sensors and laboratory-based instruments [17]. Recent research on IMS mostly focused on the improvement of the resolving power [18,19,20] and the limit of detection (LoD) [21,22,23], while leaving the dynamic range relatively less studied. Epping and Koch provided a summary of the typical dynamic range of commonly used VOC detection methods [1], in which IMS was concluded to work with low concentrations only ranging from 1 ppb to 1 ppm. However, in actual industrial production environments, the VOCs were commonly found in higher concentrations, such as tens to hundreds of ppm. Extending the dynamic range of IMS, especially to the higher end, is therefore important to make it a better tool for quantitative measurement.

## 2. The Principle of the Membrane-Based Pulsed Sampling Method

The fluctuation in humidity often interferes with the ionization process of IMS, thus affecting the response of the target analyte. To solve this problem, a semi-permeable membrane was used for sample introduction. The process of sample molecules passing through the semi-permeable membrane involves three steps. First, sample molecules are selectively adsorbed on the outer surface of the membrane. Second, the sample molecules adsorbed on the outer surface diffuse into the membrane and eventually permeate the membrane to the inner surface. Then, the sample molecules desorb from the inner surface of the membrane to the ionization chamber. The diffusion process of the sample molecules in the membrane follows Fick’s law of diffusion [24]. By choosing the material of the semi-permeable membrane, polar molecules like water can be selectively isolated from the ionization process for improved reliability of the analysis [25].

**Figure 1:**
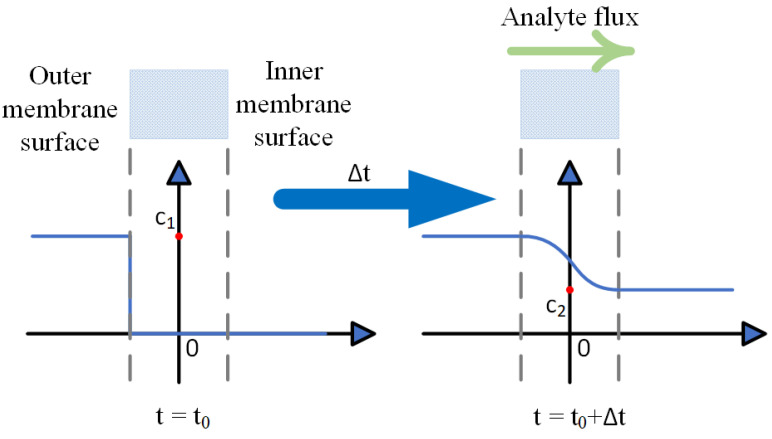
Equilibrium of analyte concentration with continuous sampling based on permeable membrane.

The direct sampling or continuous sampling method introduces a continuous flow of the gas phase sample to the outside of the membrane, thus maintaining constant concentration C_1_ of the analyte. After an interval Δt, the analyte molecules permeate the membrane through diffusion and establish an equilibrium of analyte concentration. The inlet flux of the analyte through the membrane is balanced with the removal of analyte molecules through ionization, as shown in Figure 1. In this configuration, the rate of ion generation is related to the analyte concentration C_1_ outside of the membrane by the diffusion coefficient of the analyte and the thickness of the membrane. Therefore, for a given system, the concentration of analyte C_1_ can be extracted by measuring the ion current with IMS.

The continuous sampling method relies on the removal of the analyte from the inner surface of the membrane and hence is constrained by the ionization process, which intrinsically limits the upper end of the dynamic range. Although dilution of the analyte before sample introduction can alleviate the problem, it comes with the cost of complicated hardware design. 

**Figure 2:**
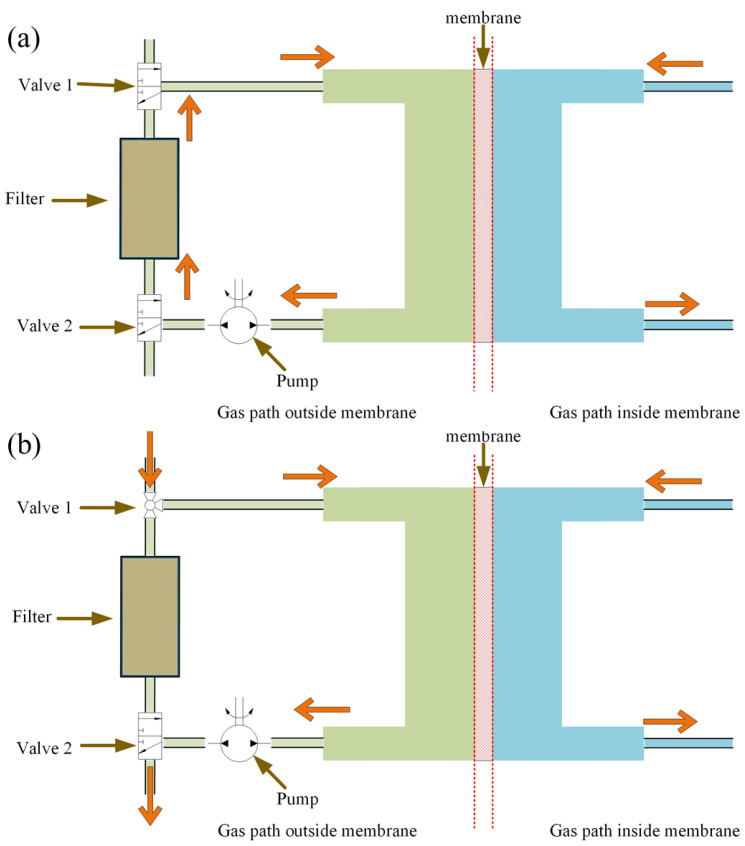
Membrane-based pulsed sampling method: (**a**) circulating mode; (**b**) sampling mode.

**Figure 3:**
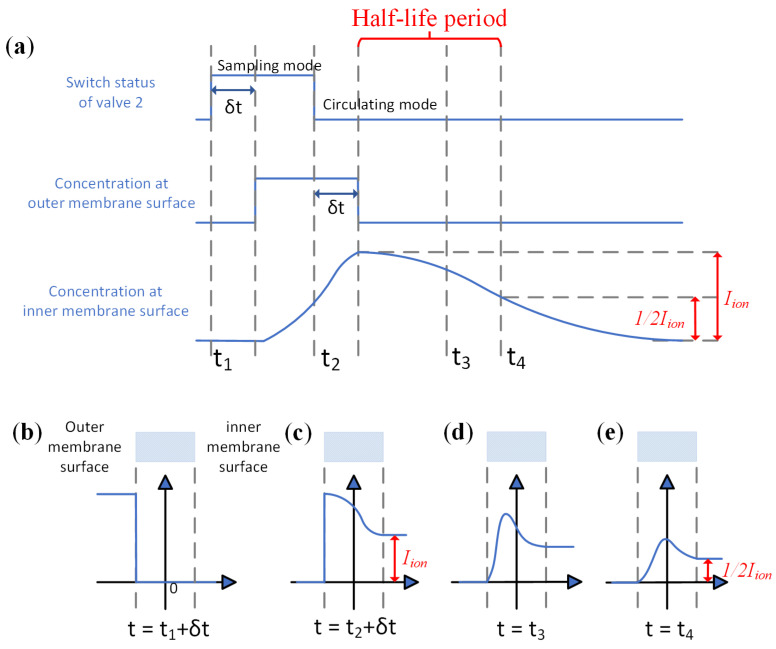
Membrane-based pulsed sampling method. (**a**) System timing and concentration changes. (**b**) Concentration distribution at moment t_1_ + δt. (**c**) Concentration distribution at moment t_2_ + δt. (**d**) Concentration distribution at moment t_3_. (**e**) Concentration distribution at moment t_4_.

We have proposed and designed a membrane-based pulsed sampling method to address the problem and extend the dynamic range of a given IMS system towards a high concentration range. As shown in Figure 2, the sampling system outside of the membrane was comprised of a sampling pump, a filter, and two valves. It can be set to two configurations, the sampling mode and the circulating mode (non-sampling mode). The working principle of our pulsed sampling system is illustrated in Figure 3. At the time of t_1_, the system was switched to sampling mode, with a fixed delay due to the dead volume of the pipe, and the analyte concentration at the outside of the membrane was brought to C_1_. After a controlled injection time (t_2_ − t_1_), the system was switched back to circulating mode. With all of the analyte adsorbed by the filter, the analyte concentration quickly decreased to 0 at the time t_2_ + δt. At this moment, the highest concentration of the analyte was inside the membrane, and the analyte in the membrane started to diffuse towards both directions, until all of the analyte molecules were eventually ionized or adsorbed. 

With given parameters of the proposed membrane-based pulsed sampling system, including the flow rate, thickness, and permeability of the membrane, the diffusion process is fixed, and the amount of analyte molecules that eventually penetrate the membrane can be correlated to the concentration of the analyte in the sample gas with a fixed relationship. In this case, for the same sample concentration, the amount of analyte to be ionized is much less than in the direct sampling method; hence, quantification of a higher concentration is possible. 

## 3. Experiment

### 3.1. Reagents and Instruments

#### 3.1.1. Reagents

N-methyl-2-pyrrolidone (NMP) is a common solvent for manufacturing positive electrodes in the battery industry. Due to its excellent chemical and thermal stability, NMP is also utilized in the production of pharmaceuticals, chemical processing, and electronics manufacturing. However, inhaling high concentrations of NMP can lead to symptoms, such as headaches, dizziness, neurological confusion, or nausea, posing significant health risks. NMP is under strict environmental scrutiny by regulatory agencies in various countries due to its hazardous effects on human health and the environment [26]. There is an urgent and realistic need to monitor the concentration of NMP in industrial production environments. In this paper, we chose NMP as the target analyte to verify the performance of the system.

The NMP used was of analytical reagent grade, with a purity greater than 99.0%, as determined through Gas Chromatography (GC), and it was purchased from Shanghai Macklin Biochemical Co., Ltd. (Shanghai, China). The molecular sieve and activated charcoal were purchased from Sinopharm Chemical Reagent Co., Ltd. (Shanghai, China). The gas sampling bag was purchased from Ningbo Hongpu Experimental Technology Co., Ltd. (Ningbo, China).

#### 3.1.2. Instruments

**Figure 4:**
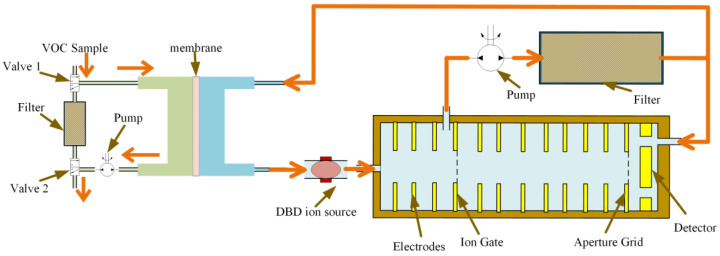
Structure illustration of the developed membrane-based pulsed sampling IMS system.

The IMS system in this paper mainly consisted of a pulsed sampler, an ionization source, an ion shutter, a drift tube, and an ion detector (Faraday plate) (Figure 4). The temperature of the membrane was set to 150 °C, and the temperature of the drift tube was set to 80 °C. Due to the significant effect of temperature on the ratio of the monomer and the dimer, we used an elevated temperature for both the membrane and the drift tube to ensure the ratio was fixed regardless of the temperature of the environment. Additionally, the half-life time is strongly dependent on the temperature, as it is ultimately based on the diffusion process of the analyte in the membrane. Compensating for the temperature effect is difficult, as the exact temperature of the membrane is hard to measure. Therefore, we chose to set the membrane at an elevated temperature to have consistent diffusion and half-life time.

The Tyndall Powell (TP) ion gate was used with a width of 0.5 mm and an opening time of 240 μs. The filed strength through the ion gate was 300 V/cm for the open state and 600 V/cm for the close state. The drift tube was 50 mm in length, and the field strength was 300 V/cm. The ring electrode for the drift tube had an inner diameter of 16 mm, and the thickness was 1.4 mm. The thickness of the insulating ring was 1.2 mm, and the inner diameter was 18 mm, which was slightly larger to minimize parasitic capacitance and charge accumulation on the insulating surface. The drift tube ended with an apertures grid at 1 mm in front of the faraday plate, and the voltage was set to 60 V.

As the ionization source we employed in this work was dielectric barrier discharge, the humidity significantly affects the ionization process and, therefore, the measurement. The use of a filter including a molecular sieve and activated charcoal for the circulating gas inside the membrane was the key to maintaining the required humidity in the ionization chamber and the reproducibility of the measurement. The use of a semi-permeable membrane also helped with the reproducibility, as most polar molecules, including water, are kept outside. The carrier gas used in the system was clean air filtered with activated carbon and molecular sieves, with a dew point maintained below −40 °C.

The injection time is defined as t_2_ − t_1_ in Figure 3 and set to 500 ms for all experiments. Reducing the injection time could further enhance the dynamic range, but with two limitations. First, the accuracy of the switching valve we used was 10 ms, which means that with a shorter injection time, the uncertainty of the analyte introduced into the system was significant. Second, as the analyte molecules were introduced into the gas pipe, diffusion also occurred alongside flow movement. If the injection time is too short, then by the time the analyte reaches the membrane, the concentration would significantly change. Taking those two considerations together, we chose 500 ms as a reasonable trade-off, which is adequate to demonstrate the effect of this novel method rather than obtaining the very best performance. The measurement period was set to 240 s to better eliminate the interference of contamination resulting from the measurement of the high-concentration analyte. Sacrificing a certain degree of real-time responsiveness was unavoidable when aiming to enhance the dynamic range of the system. The flow rate of the sample pump was set to 280 mL/min, which meant approximately 2.33 mL of the sample gas was taken in for each injection. The inner diameter and length of all of the gas line pipe were kept as short as possible, with an estimated dead volume of 0.85 mL, giving a delay of 182 ms. The half-life period was defined as the time for the height of the measured ion peak to decrease to half of its maximum value during each measurement. 

### 3.2. Sample Preparation

The sample of the diluted analyte in the gas phase was prepared through volatilization by calculating the amount of the liquid phase analyte in the corresponding volume of filtered clean air in a Polytetrafluoroethylene (PTFE) bag. All experiments were carried out under an atmospheric pressure at 20 °C to avoid condensation.

## 4. Results and Discussion

### 4.1. Discussion of Ion Peak Positions

**Figure 5:**
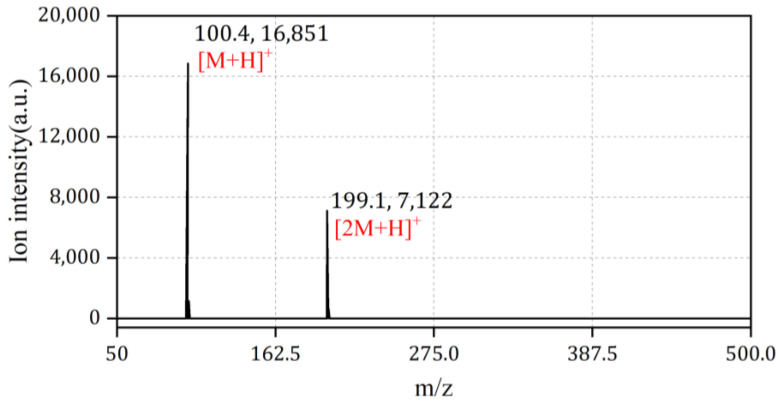
Spectrum measurements of NMP using MS.

The NMP sample was firstly characterized with an ion trap mass spectrometer equipped with an atmospheric ionization source, as shown in Figure 5. Two distinct ion peaks can be observed with a mass-to-charge ratio (*m*/*z*) of 100 and 199, corresponding to the monomeric dimeric molecular product ion of NMP, denoted as [M + H]^+^ and [2M + H]^+^, respectively. The presence of the dimer ion peak indicates excessive analyte molecules in the ionization reaction, such that two protonphilic NMP molecules share one positive charge.

**Figure 6:**
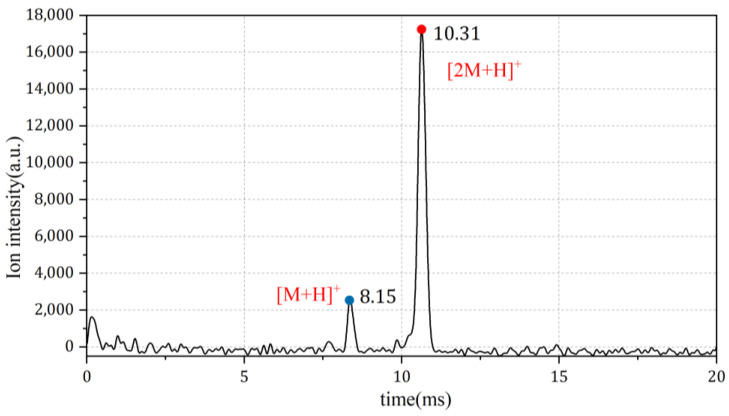
Spectrum measurements of NMP using IMS.

Similar observations can be made based on the IMS spectrum (Figure 6). The monomeric ion [M + H]^+^ appears at 8.15 ms on the spectrum, and the dimeric ion [2M + H]^+^ is detected at 10.31 ms. By varying the concentration of NMP, a clear trend of the relative amplitude of the monomer and dimer peaks can be observed. Starting from a low concentration, the monomer peak appears first and gets higher as the concentration increases, until the dimer peak appears. As the intensity of the dimer ion increases, the intensity of the monomer ion gradually decreases, until, eventually, the monomer peak disappears, leaving only the dimer peak visible in the spectrum.

### 4.2. Discussion of Continuous Sampling Results

**Figure 7:**
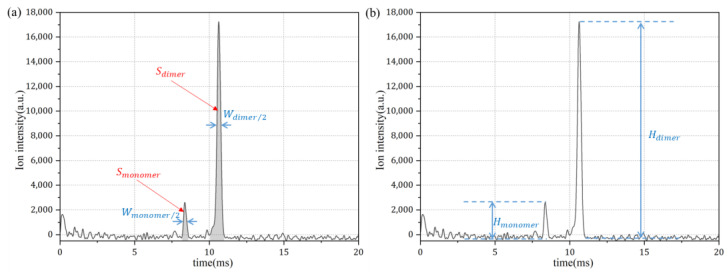
Two methods for calculating concentration in continuous sampling mode. (**a**) The area of the corresponding peak. (**b**) The height of the corresponding peak.

With the continuous sampling method, the concentration of the analyte can be calculated from the spectrum with two methods, with either the height or the area of the corresponding peak, as shown in Figure 7.

The peak area can be calculated using Equation (1):

(1) $$\left\{\begin{array}{c} \begin{array}{c} S_{monomer}=W_{monomer/2}*H_{monomer} \end{array}\\ S_{dimer}\;\;=W_{dimer/2}*H_{dimer}\\ S_{total}=S_{monomer}+2*S_{dimer} \end{array}\right.$$

**Figure 8:**
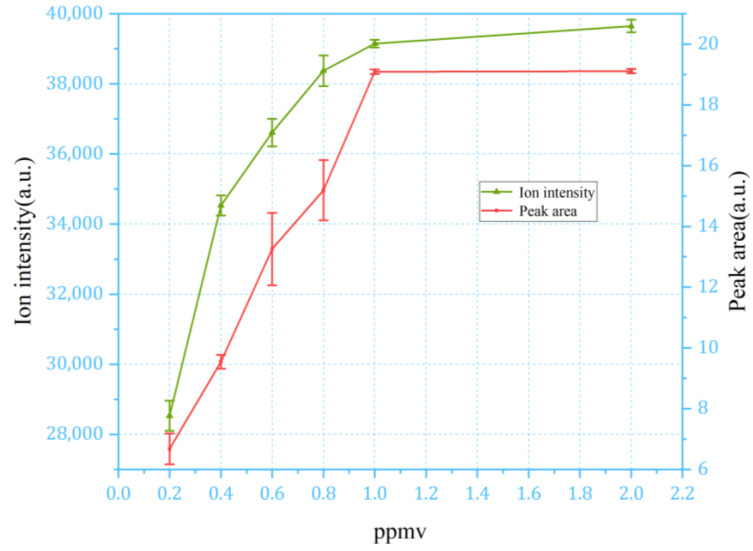
The concentration curve of NMP with a concentration range of 0.2 to 2 ppm in continuous sampling mode.

The parameters of Equation (1) are as follows. $S_{monomer}$ represents the area of the monomer peak, $W_{monomer/2}$ denotes the half-peak width of the monomer peak, and $H_{monomer}$ indicates the peak height (ion intensity) of the monomer. Similarly, $S_{dimer}$ is the area of the dimer peak, $W_{dimer/2}$ is the half-peak width of the dimer peak, and $H_{dimer}$ represents the peak height (ion intensity) of the dimer. $S_{total}$ is the sum of the monomer peak area and two times the dimer peak area, as each positive ion arriving to the detector represents two analyte molecules within the dimer peak. The same consideration applies to the calculation based on peak height, as well, as the ion intensity of the dimer peak must be doubled to calculate the corresponding concentration. The measured calibration curve of NMP with a concentration range of 0.2 to 2 ppm is given in Figure 8.

It can be observed that with the continuous sampling method, the response of the IMS signal saturates at the NMP concentration of around 1 ppm. Both characterization methods demonstrated a similar dynamic range, with ion intensity (peak height) giving the smaller error bar. Moreover, the calculation of ion intensity is relatively easier than the peak area, offering advantages for data processing and algorithm design. Therefore, the intensity of the ion current was adopted for measuring the analyte concentrations using the continuous sampling method. 

### 4.3. Discussion of Pulsed Sampling Results

**Figure 9:**
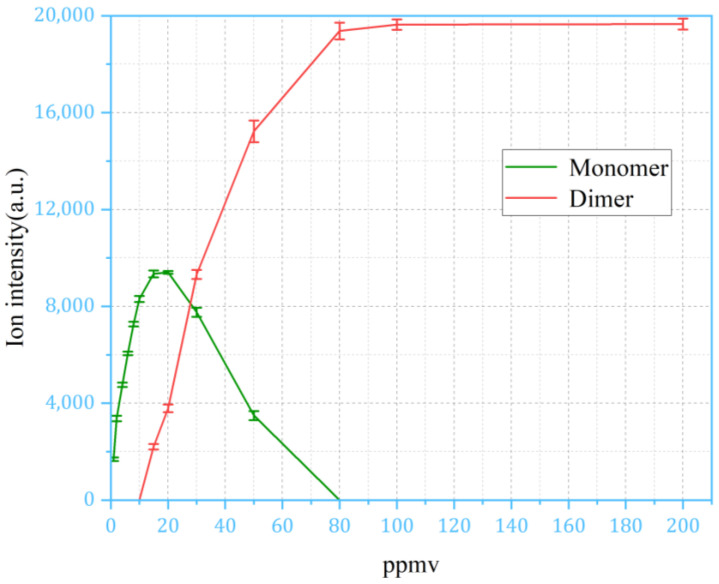
The relationship between the peak heights of monomers and dimers of NMP and their concentrations ranging from 1 ppm to 200 ppm in the membrane-based pulsed sampling method.

**Figure 10:**
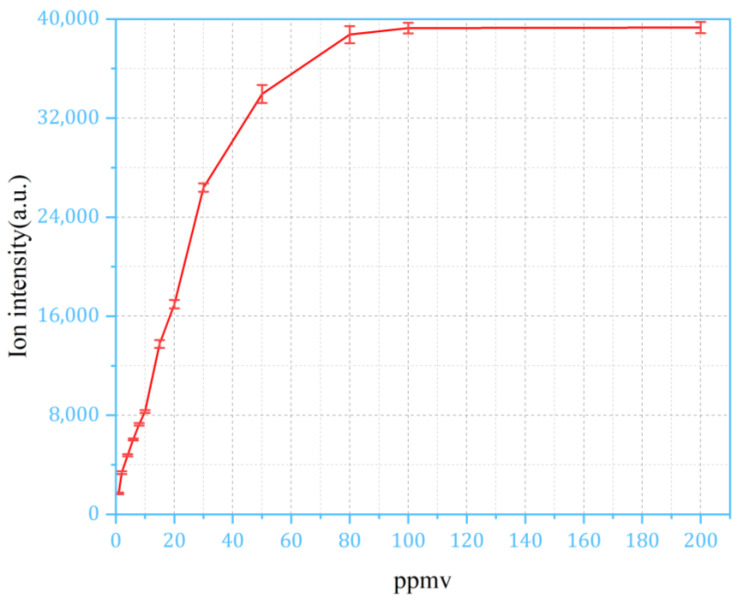
The results calculated based on the height of the corresponding peak in the membrane-based pulsed sampling method.

For the continuous sampling method, the IMS response of NMP saturates at 1 ppm; to extend the dynamic range, the response of the IMS signal must be examined with the pulsed sampling method at high concentrations. The plot in Figure 9 uses the peak height (maximum value) after sampling. As in Figure 9, the peak height of the monomer and dimer of NMP was plotted in relation to concentrations ranging from 1 ppm to 200 ppm. It can be observed that the relationship between the monomer and the dimer peak follow the same trend at the concentration range between 1 ppm and 80 ppm, until the monomer peak disappears and the dimer peak saturates. Figure 10 is calculated based on the values in Figure 9, following the method described in Figure 7. In the pulsed sampling method, the ion intensity demonstrates good response characteristics within the concentration range of 1 ppm to 80 ppm.

**Figure 11:**
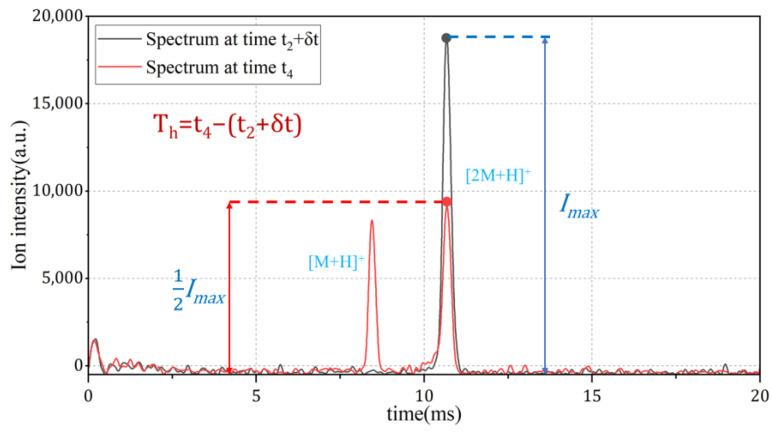
Method for calculating half-life period.

**Figure 12:**
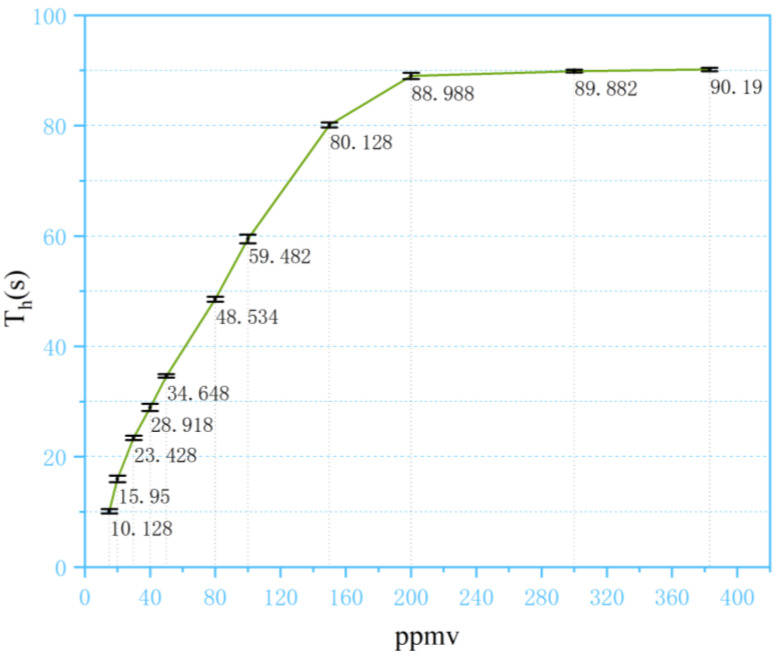
Relationship between T_h_ and concentration in membrane-based pulsed sampling method.

In case of concentrations higher than 80 ppm, a different indicator is required. Considering the diffusion process of the analyte, as shown in Figure 3, although the highest concentration at the inner side of the membrane stays the same, the amount of analyte molecules injected into the membrane is affected by the concentration at the outer side of the membrane. When the sampling pulse finishes, the analytes within the membrane start to diffuse to both directions, and the time constant of this diffusion process relies on the amount of analyte molecules inside the membrane and, therefore, the initial concentration of the sample gas. As illustrated in Figure 11, the half-life period of NMP, denoted as T_h_, which is the time it takes for the dimer peak to diminish to half of its highest level, shows that a correlation between T_h_ and the concentration of the analyte in the sample gas can be established, as shown in Figure 12.

Taking T_h_ as a measure of sample concentration, the dynamic range was effectively extended to around 200 ppm, with an improvement of two orders of magnitude compared to the conventional continuous sampling method. From Figure 12, it can be found that with concentrations higher than 200 ppm, T_h_ did not respond to the change in sample concentration. Based on experiments with other substances, the detector can detect an ion intensity of approximately 25,000, indicating that the detector has not reached saturation. It is assumed that this is because the amount of analyte that can be injected into the membrane is eventually limited by the permeability of the membrane material. With higher concentrations, even if the concentration gradient is larger, the membrane cannot intake more analyte molecules. When the analyte was removed from the outer side of the membrane, the same amount of analyte molecules stored in the membrane ended up with the same T_h_. In this case, we tested the saturated vapor at 20 °C, corresponding to the concentration of 383 ppm, and the response of T_h_ stayed relatively the same as that of 200 ppm.

### 4.4. Integration of Sampling Method for Extending Dynamic Range

**Figure 13:**
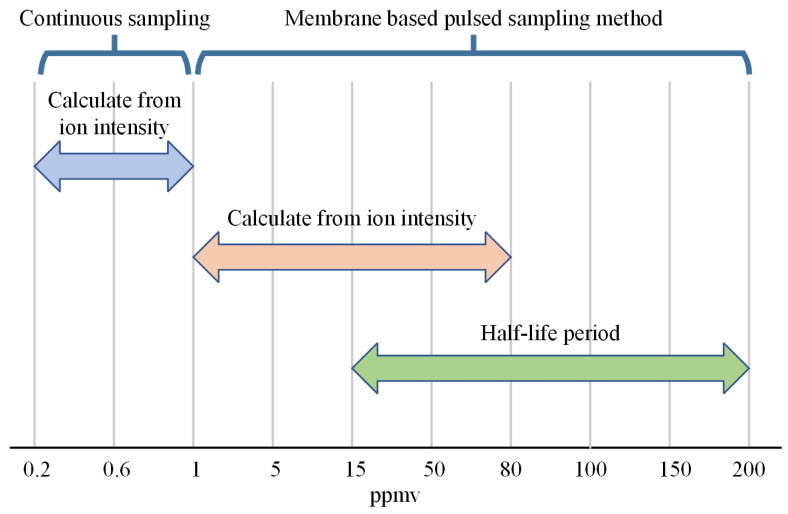
Summary of different sampling and characterization methods for the measurement of NMP at varying concentration levels.

Although the proposed pulsed sampling method offers the ability to measure high concentrations, it is always desired to have an extended dynamic range covering both low and high concentrations. Figure 13 provides a detailed comparison of different sampling and characterization methods for the measurement of NMP at varying concentration levels. Continuous sampling provides real-time monitoring capabilities, but it is limited to a dynamic range of only 0.2 ppm to 1 ppm. Moreover, when the system collects samples with higher concentrations, the cleaning time required for the system is prolonged, posing significant challenges for subsequent measurements. The membrane-based pulsed sampling method increases the measurement upper limit from 1 ppm to 200 ppm. 

Combining the methods described in Figure 13 can offer an extended dynamic range of three orders of magnitude, from 0.2 ppm to 200 ppm. Due to the implementation of a new sampling method without altering the structure of the IMS, the selectivity and sensitivity remain the same, and the resolving power and amplification stay the same.

**Figure 14:**
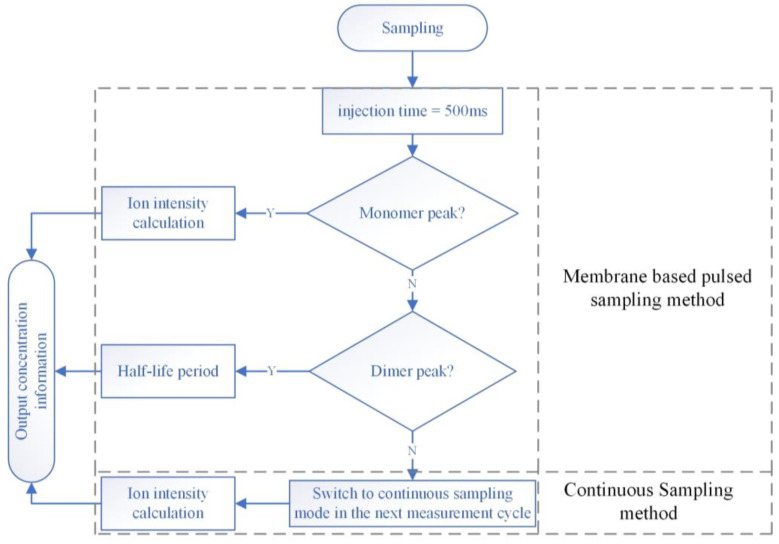
Integrated measurement strategy based on control software.

A measurement strategy was developed together with the control software to realize this extended dynamic range on a single instrument, as illustrated in Figure 14. It is worth noting that the hardware configurations for continuous and pulsed sampling are fully compatible. The switching of the two sampling modes, and also the characterization method, can all be performed through software control automatically.

## 5. Conclusions

In this work, we have proposed and developed a novel sampling method, together with characterization indicators, to enable the application of IMS for higher concentrations. Taking NMP as example, the upper limit of measurement was improved from 1 ppm to 200 ppm. The working principle of this new method was analyzed and examined. Moreover, a measurement strategy was designed and implemented to integrate this proposed pulsed sampling method with the conventional continuous sampling method, resulting in a single IMS instrument with the ability to perform quantitative analysis of over three orders of magnitude. 

Future work will include detailed mathematical modeling of the sampling methods, validation of the measurement strategy and the developed instrument with different types of VOCs, and further optimization of the system for better performance.

## Data Availability

Data are contained within the article.

## References

[1] Epping R., Koch M. (2023). On-Site Detection of Volatile Organic Compounds (VOCs). Molecules.

[2] Reimann S., Lewis A.C. (2007). Anthropogenic VOCs. Volatile Organic Compounds in the Atmosphere.

[3] Mizukoshi A., Kumagai K., Yamamoto N., Noguchi M., Yoshiuchi K., Kumano H., Yanagisawa Y. (2010). A novel methodology to evaluate health impacts caused by VOC exposures using real-time VOC and Holter monitors. Int. J. Env. Res. Public. Health.

[4] Rumchev K., Brown H., Spickett J. (2007). Volatile organic compounds: Do they present a risk to our health?. Rev. Environ. Health.

[5] Koppmann R. (2007). Volatile organic compounds in the atmosphere: An overview. Volatile Organic Compounds in the Atmosphere.

[6] Verma G., Gokarna A., Kadiri H., Nomenyo K., Lerondel G., Gupta A. (2023). Multiplexed Gas Sensor: Fabrication Strategies, Recent Progress, and Challenges. ACS Sens..

[7] Blanco-Rodriguez A., Camara V.F., Campo F., Becheran L., Duran A., Vieira V.D., de Melo H., Garcia-Ramirez A.R. (2018). Development of an electronic nose to characterize odours emitted from different stages in a wastewater treatment plant. Water Res..

[8] Park S.J., Park C.S., Yoon H. (2017). Chemo-Electrical Gas Sensors Based on Conducting Polymer Hybrids. Polymers.

[9] Nath N., Kumar A., Chakroborty S., Soren S., Barik A., Pal K., de Souza F.G. (2023). Carbon Nanostructure Embedded Novel Sensor Implementation for Detection of Aromatic Volatile Organic Compounds: An Organized Review. ACS Omega.

[10] Poole C.F. (2013). Alkylsilyl derivatives for gas chromatography. J. Chromatogr. A.

[11] Santos F.J., Galceran M.T. (2002). The application of gas chromatography to environmental analysis. TrAC Trends Anal. Chem..

[12] Lim Y.M., Swamy V., Ramakrishnan N., Chan E.S., Kesuma H.P. (2023). Volatile organic compounds (VOCs) in wastewater: Recent advances in detection and quantification. Microchem. J..

[13] Li T., Zhu X., Hai X., Bi S., Zhang X. (2023). Recent Progress in Sensor Arrays: From Construction Principles of Sensing Elements to Applications. ACS Sens..

[14] Ziyatdinov A., Marco S., Chaudry A., Persaud K., Caminal P., Perera A. (2010). Drift compensation of gas sensor array data by common principal component analysis. Sens. Actuators B Chem..

[15] Wilson A.D. (2012). Review of Electronic-nose Technologies and Algorithms to Detect Hazardous Chemicals in the Environment. Procedia Technol..

[16] Lee J., Sayler S.K., Zhou M., Zhu H., Richardson R.J., Neitzel R.L., Kurabayashi K., Fan X. (2018). On-site monitoring of occupational exposure to volatile organic compounds by a portable comprehensive 2-dimensional gas chromatography device. Anal. Methods.

[17] Costanzo M.T., Boock J.J., Kemperman R.H.J., Wei M.S., Beekman C.R., Yost R.A. (2017). Portable FAIMS: Applications and Future Perspectives. Int. J. Mass. Spectrom..

[18] Williamson D.L., Nagy G. (2022). Evaluating the Utility of Temporal Compression in High-Resolution Traveling Wave-Based Cyclic Ion Mobility Separations. ACS Meas. Sci. Au.

[19] Conant C.R., Attah I.K., Garimella S.V.B., Nagy G., Bilbao A., Smith R.D., Ibrahim Y.M. (2020). Evaluation of Waveform Profiles for Traveling Wave Ion Mobility Separations in Structures for Lossless Ion Manipulations. J. Am. Soc. Mass. Spectrom..

[20] Ahrens A., Hitzemann M., Zimmermann S. (2019). Miniaturized high-performance drift tube ion mobility spectrometer. Int. J. Ion. Mobil. Spectrom..

[21] To K.C., Ben-Jaber S., Parkin I.P. (2020). Recent Developments in the Field of Explosive Trace Detection. ACS Nano.

[22] Li M., Wang S., Xu C., Ruan H., Wang W., Chen C., Li H. (2021). Parallel Coupling of Ion Mobility Spectrometry and Ion Trap Mass Spectrometry for the Real-Time Alarm Triggering and Identification of Hazardous Chemical Leakages. Anal. Chem..

[23] Fisher D., Lukow S.R., Berezutskiy G., Gil I., Levy T., Zeiri Y. (2020). Machine Learning Improves Trace Explosive Selectivity: Application to Nitrate-Based Explosives. J. Phys. Chem. A.

[24] Bell R.J., Short R.T., van Amerom F.H.W., Byrne R.H. (2007). Calibration of an In Situ Membrane Inlet Mass Spectrometer for Measurements of Dissolved Gases and Volatile Organics in Seawater. Environ. Sci. Technol..

[25] Eiceman G.A., Karpas Z., Hill H.H. (2016). Ion Mobility Spectrometry.

[26] Sliz R., Valikangas J., Silva Santos H., Vilmi P., Rieppo L., Hu T., Lassi U., Fabritius T. (2022). Suitable Cathode NMP Replacement for Efficient Sustainable Printed Li-Ion Batteries. ACS Appl. Energy Mater..

